# Seamless Crowns

**Published:** 1904-05-15

**Authors:** M. L. Ward


### SEAMLESS CROWNS.

BY M. L. WARD, D.D.S.

Read before the Central and Southwestern Michigan Dental Societies, April 12, 1904.

Owing to the existing objections to seamless gold crowns, it gives me some embarrassment to attempt to satisfactorily present the subject upon short notice. It is to be regretted, but it is true nevertheless, that many of those who are the the most persistent in objecting, have a wrong conception of the word seamless, and look upon the subject of seamless gold crowns as though they were "poor fit" gold crowns. It will become apparent as we progress with a comparison of the older with the newer methods that this was once partially true, and that up-to-date methods are radical departures from the methods heretofore employed, and overcome the principal objections to seamless crowns, giving in almost every case the most satisfactory results. The requirements of all artificial crowns are that they should as nearly as possible restore the appearance and function of the natural teeth, and afford no opportunities for disease-producing agencies. To detail the requirements we would say that they should restore contact points, anatomical contour, occlusion and produce no irritation in their attachment. It is obvious that color is a requirement which can not be met, so for this reason we should avoid all gold crowns on any root anterior to the first molar, with the exception of the lower bicuspids in cases where they do not show. It is a rumor that has been erroneously handed along, since the invention of the Morrison and older outfits, that the principal defects in seamless crowns were their inability to meet the requirements of abnormally-shaped roots and anomalous articulations. It is a common observation that many, perhaps a great majority, of these crowns are imperfectly fitted, or the roots are imperfectly prepared, and an instrument passed around their borders discovers the existence of an irregular shoulder produced by lack of adaptation of the band, or because the sectional area at the gum margin was greater than beneath it. The evils attending and following the placing of this class of crowns are due almost entirely to the operator, and are found not only on roots having seamless crowns, but upon those having soldered ones as well. It is a task of some little difficulty to properly shape a root for the reception of a collar crown, and no easy one to accurately fit a metallic band to the prepared root. The shape of the root seems to have been well understood by the older men of the profession and taught by most of them, but it is a thing so easily neglected that a review of the process as given by one of our older authors may help to impress upon the profession the necessity of having a properly-prepared root before attempting to take a measurement for a cap crown. The process of shaping the surfaces of natural crowns or roots for the different styles of artificial crowns requires that the greatest diameter of the crown or root be just underneath the gum margin, and that the sides of the remaining portion of the tooth be reduced sufficiently to admit any peripheral measurement that may be taken at this place to pass freely to the occlusal surface. Sometimes this is already the case, but there is not room to perfectly adapt a measurement and much less a band for an artificial cap crown. In such cases, there should be enough tooth substance removed to make space for the material used to pass freely between the tooth to be crowned and the adjacent teeth, and enough from the cervix at the junction of the enamel and dentine, so that all sides are level and parallel with the line of the root. Such a form is necessary to admit of a perfect adaptation of any collar band or ferrule, as they are variously designated, that we may employ in the construction of a cap crown, whether it be soldered or seamless or any combination of either with porcelain, requiring a band around the root. Having prepared the root in this way, we may have overcome at least one of the difficulties that perplexes the novice of to-day, who undertakes to make seamless crowns from tooth forms, natural teeth, etc., and slip them over teeth or roots whose greatest peripheral measurement is above the gum margin. It is evident that this can not be successfully done. It needs no discussion. There are several methods of constructing seamless gold crowns, many of which are so limited in sizes and methods of construction and adaptation that they are of little value to a general practitioner. One of the older appliances was known as the Morrison. With this, as with the newer ones, it consisted of a series of holes arranged consecutively in a flat piece of steel, as a draw plate for making the shells. For finishing the shape of the crown, there was a die plate of little more than ordinary depth, into which the shell was stamped with a soft wood stick, or a stick and a small amount of rubber. It can be plainly seen that it was very difficult, if not impossible, to get a contact point, contour or to properly change the cusps when it was needed to make an occlusion. It is a crude process to rely upon in a majority of cases. For years seamless shells have been swaged over metal tooth forms, and if any attempt was made to contour the crowns fusible alloy was used and the finished crown relieved of the metal core by heating to the melting point of the alloy. The difficulty of contracting the shell at the cervix and forming the metal die were the undesirable features of this method. Another outfit known as the Berry was an improvement in some ways, but a failure in others, so that it gained disfavor in many places. The draw plate was quite a success, but the method of converting the shell into a crown was not so satisfactory, as many of you know who have tried to obtain counter-dies by the dipping process. Many of the newer outfits, such as the Sharp, and Kidder, produce beautiful shells, but fail to show much mprovement in the way of converting them into crowns. The draw plates seem to have been given more attention than any other part of the outfit and as a result we have one or two on the market that, with one exception, are nearly perfect. It is here that I want to continue my crusade upon the manufacturers that I began nearly three years ago when I had a thirty-gauge outfit ground to a twenty-eight gauge. Since that time I have had several other outfits ground for students of the 1902-03 classes who are ardent admirers of a perfectly contoured crown, and have yet to hear from one of them who is not making a decided success of seamless crowns. It is not all due to the gauge of the gold however, but I attach a great deal of importance to this feature and have insisted upon a twenty-eight gauge outfit in preference to a thirty gauge every time I had advised any one to buy one, and have as many times met with the most persistent objections from the dealers founded upon nothing more than economy. It may be well to mention the fact here that all makes of gold can not be successfully used in making seamless crowns, some of it being very ductile, while other makes are so much different that it is almost impossible to draw a shell into a crown. The devices for converting the shell into a crown, sent out with most of the outfits are very crude affairs when compared with the elaborate draw plates accompanying them. It is here that I want to show you another of the causes of so many seamless crown outfits being on on laboratory shelves, and hope to be able to convince you at my clinic to-morrow afternoon that I am right. To describe and illustrate the process of making a seamless crown from a tooth form, which is not an undesirable way when done by skillful operators, let us take one of the most difficult crowns to construct anatomically correct —an inferior molar or bisuspid.) A wire measurement is taken of the root. This is compared with the different forms for this place, noting their general shape and cervical measurement, bearing in mind that the cervix of the gold crown should preferably be smaller than larger, as it can be easily expanded while its contraction is difficult. It is not essential that the curve of the tooth form should correspond with the root to be crowned, because the gold will readily take the proper shape as the crown is adjusted. From this form a sectional mold or counter-die is made by pouring the fusible allow directly over the tooth form. Into the counter-die a gold shell is adjusted fitting tightly the orifice of the closed counter-die. The shell is expanded to the general form of the counter die with a small amount of vulcanite, a stiff piece of wood and a mallet, facilitating the operation by frequently opening the counter-die and annealing the gold in alcohol. Such a crown may be lengthened at any of its cusps or margins to form any occlusion needed by cutting out the metal with a bur or excavator and reswaging. The same process exactly will restore contact points, while to reduce its length the mallet and plugger are all that is needed. It will be seen that there are some undesirable features to this process, but a very prettily-contoured crown may be made in this way and just as good a fit at the cervical portion as with any soldered crown, if care has been taken to select a small enough tooth form. It is not an undesirable method in the hands of skillful operators and enables them to do much better shaped crowns than can otherwise be made. Indeed, I think the operator who makes a poor fit with this style of crown will with a soldered one as well. The method I use most, and feel confident that meets the requirements more nearly in a majority of cases, is the natural-bite method. By this I mean a crown made from a tooth form that was carved to fit the place we desire to occupy with a gold crown. This may be accomplished in more than one way, but to accomplish it quickly and accurately enough to make its use desirable to a general practitioner, we may proceed somewhat as follows:

For a band, cut a strip of copper of the required length and width, which may be ascertained by a wire measurement and an adaptation of the band a few times. If a lap-joint is to be made, the end to form the underlap is beveled with a file, but if a butted joint is preferred, the ends are made as straight and smooth as possible. The strip is then bent with suitable pliers to the average form, noting any deviation from such average and to the cervical periphery of the root to be crowned. It is then placed on the root and adapted as closely as possible, letting the edge of the metal pass under the free edge of the gums. Remove it and cut off the excess in length and width, continuing the adaptation and contouring until it fits the root and approximates the cervical portion of an anatomically-correct tooth. At the last adjustment to the root the lap-over is marked on the metal with a pointed instrument and the joint made at this mark, using as little solder as possible. The band may now be placed on a small anvil, and the joint tapped down and trimmed level and smooth. When the band is formed, it is adjusted on the root, and pressed up to or just under the gum margin as you choose, aided by a piece of wood and a light mallet, or such devices as you are accustomed to using. A line parallel with the gum margin is marked with a pointed instrument on the band, which is then removed, trimmed, re-adjusted and marked again, continuing the process until the band fits to or just under the gum margin and reaches one-third of the distance to the occlusal surface. Having adjusted the collar to the root with a metal much more pliable than 22-karat gold, we have reduced inaccuracy of fit to a minimum With the band in place, we now place upon the tooth to be crowned a small amount of impression compound, which has been over-heated somewhat, or a small amount of plaster containing about one-fourth whiting, and require the patient to place the teeth in their natural occlusion, thus securing accurately the bite and length of the crown, no matter how irregular it may be. After allowing the compound to harden, or the plaster to set, it is removed together with the band. To prevent the impression material and band from separating, melt a little sticky wax inside the band with a spatula. The impression material can now be carved to represent as nearly as possible a natural tooth, without destroying the part necessary to perform an occluding surface with the antagonizing teeth. Having done this properly, we have an exact model of the crown we wish to make. It will be noticed by careful observation of crowns made by any natural-bite method, that different operators differ widely as to what a properly carved crown is. Many of them will obtain a bite and then carve it to represent a natural tooth, thus destroying the greater part of the occluding surface. In many cases this is wrong, and is especially so in many of cases where extractions have been made. What is the object of the gold cap crown, if it is not to restore a natural or provide an artificial occlusion? If we prefer form to occlusion, especially when it is at the expense of the latter, then we may as well use tooth forms, natural teeth or swage the cusps in a die plate. Having carved the impression material to the shape of the crown desired, make a counter-die of fusible alloy, preferably in the three-part flasks, originally designed by Dr. William H. Dorrance, which, through a misunderstanding, was recently advertised under my name. Its use I will demonstrate in the clinic and trust I may be able to show you that it is equally as necessary to insure perfect success with seamless crowns as is a properly prepared tooth form or the gauge and kind of gold.

The alloy used to form the counter-die should be one with a melting point below 170 degrees Farenheit, whether using compounds or plaster, and should be poured as cool as possible and cooled in water as soon as it is set, if you are using compound to obtain a bite. The sectional counter-die thus obtained is an accurate outside measurement of the crown we are to construct, without a step between the construction of the form and its reproduction in gold. Into this sectional counter-die a twenty-two karat gold shell of the same gauge as the copper used to form the band, and made preferably from one of the latest draw plates, is driven well down in to the counter-die and swaged with vulcanite and a hard piece of wood until it fills every part of the counter die. Many devices in the form of pliers hasten the changing of the cusps when it is found necessary in adjusting the crown before filling the cusps with solder, but its skillful performance can be attained as well with a mallet and plugger if the pliers are not at hand. Many operators complain about the apparatus necessary to construct seamless gold crowns, while others do very good work in an easy manner with the screw presses, telescoping devices and the like. The same is true with other crown construction where experts in crown and bridge-work succeed, using no appliances but pliers and shears guided by an intuitive perception of the requirements in each case. I do not wish to invite controversy, and am reluctant to depreciate other crowns, but my experience with this style of crown warrants me in making the claim for it, devoid of any incentive whatever for exaggeration, that it represents as nearly as is practicable the anatomical contour—it gives a perfect occlusion and perfect contact points. It presents a uniform surface of high karat gold, is easily and quickly adjusted and requires little polishing.

### DISCUSSION.

D. N. S. Hoff: Former methods of constructing the seamless gold crown were so faulty that this style of crown largely fell into disuse with conscientious dentists and the tendency was to go back to the soldered crown. Recent appliances and methods have, however, again brought the seamless crown to the front, and it is now being made more acceptably than ever before.

The method described by Dr. Ward is one of gradual evolution and has passed through so many hands that it would be difficult to say to whom belongs the credit for its invention. The advantage of the method described by Dr. Ward is that it makes the fitting of the root as nearly perfect as it is possible to hope for, and allows for shaping and contouring the crown to individual cases with a precision that produces both useful and artistic results. By using a soft copper band for the matrix, it is possible to determine with ease the shape and extent of the stump fitting. The body of the crown can also be molded to such form as will harmonize with the adjoining teeth, and by using a plastic material an exact and comfortable occlusal surface is secured, which provides for complete masticating function. The reproduction in gold of the completed form is so easily accomplished with the three-part Dorrance flask and the fusible alloy that it is certainly a most desirable method of producing all gold seamless crowns. The present notion that these crowns can be made of thin metal is erroneous and must be changed. They should not be made of less than twenty-eight gauge metal and the occlusal portion should be strengthened with solder or fused plate.

Dr. C. H. Warboys: I used the seamless crown as formerly made for several years and abandoned it because results were not satisfactory. I am now making them by better methods and am satisfied that they are a much better crown than the soldered crown. I use the thirty-gauge Berry shell machine, but I start with a twenty-eight guage plate and can do this by using a size smaller punch than is indicated until I get to the last one. This carries my shell through to the drawing in the last hole as twenty-gauge and it is then drawn through the last time with the punch appropriate to the hole, and this draws the sides of the shell down to thirty-gauge and leaves the cap twenty-eight gauge. If the model is built up to the natural bite, there is no need to strengthen it with solder. I use cement for getting the bite instead of modeling compound as Dr. Ward does. The cement is harder and can be polished so that it makes a nice, smooth matrix in the fusible alloy, and your crown comes out smooth and polished.

Dr. C. F. Lauderdale: I am not making seamless crowns now, largely because I have not used the methods here advocated which it seems to me most excellent. There is an opportunity for improvement in this class of crowns from the artistic standpoint. By methods formerly used to produce these crowns they could not be made to imitate the forms of the natural teeth, and were, because of lack of proper occlusion, as useless as inartistic. If their adaptation can be confined to suitable locations in the mouth, I have no doubt they may be put to good use in many cases.

Dr. Ward: Any improvement over the method I have advocated must be along the line of more artistically carving the occlusal and lateral surfaces of the model, as a method of securing more accurate fit of the stump and gum festoon can not be expected, since this method gives exactness, and nothing more is necessary. I prefer a hard modeling compound to get bite length and occlusal markings, as it can be carved away or built up at pleasure, and can be made sufficiently smooth to produce practically a polished metal matrix into which to swage the gold shell, Of course it is to be expected that any method, however good it may be, in the hands of thoughtless or inefficient persons can be perverted and made to yield bad results. It may even be used for unprofessional purposes, such as flimsy bridge-work, or crowns for the conspicuous anterior teeth. The possibilities of this method legitimately used, it seems to me ought to make it worth every one's while to become acquainted with its details, as he certainly will find many cases of treatment will yield to its good offices.

